# Strengthening Mentoring in Low- and Middle-Income Countries to Advance Global Health Research: An Overview

**DOI:** 10.4269/ajtmh.18-0556

**Published:** 2018-11-14

**Authors:** Andres G. Lescano, Craig R. Cohen, Tony Raj, Laetitia Rispel, Patricia J. Garcia, Joseph R. Zunt, Davidson H. Hamer, Douglas C. Heimburger, Benjamin H. Chi, Albert I. Ko, Elizabeth A. Bukusi

**Affiliations:** 1 Emerge, Emerging Diseases and Climate Change Research Unit, School of Public Health and Administration, Universidad Peruana Cayetano Heredia, Lima, Peru;; 2 University of California Global Health Institute, San Francisco, California;; 3 St. John’s Research Institute, Bangalore, India;; 4 Centre for Health Policy and Research Chair, School of Public Health, University of the Witwatersrand, Johannesburg, South Africa;; 5 Epidemiology, Sexually-Transmitted Infections and Human Immunodeficiency Virus Unit, School of Public Health and Administration, Universidad Peruana Cayetano Heredia, Lima, Peru;; 6 Departments of Neurology, Global Health, Epidemiology and Medicine (Infectious Diseases), University of Washington, Seattle, Washington;; 7 Department of Global Health, Boston University School of Public Health, Boston, Massachusetts;; 8 Vanderbilt Institute for Global Health, Nashville, Tennessee;; 9 University of North Carolina, Chapel Hill, North Carolina;; 10 Department of Epidemiology of Microbial Diseases, Yale School of Public Health, New Haven, Connecticut;; 11 Instituto Gonçalo Moniz, Fundação Oswaldo Cruz, Salvador, Brazil;; 12 Research Care Training Program, Center for Microbiology Research, Kenya Medical Research Institute, Nairobi, Kenya

## Abstract

Mentoring is a proven path to scientific progress, but it is not a common practice in low- and middle-income countries (LMICs). Existing mentoring approaches and guidelines are geared toward high-income country settings, without considering in detail the differences in resources, culture, and structure of research systems of LMICs. To address this gap, we conducted five Mentoring-the-Mentor workshops in Africa, South America, and Asia, which aimed at strengthening the capacity for evidence-based, LMIC-specific institutional mentoring programs globally. The outcomes of the workshops and two follow-up working meetings are presented in this special edition of the *American Journal of Tropical Medicine and Hygiene*. Seven articles offer recommendations on how to tailor mentoring to the context and culture of LMICs, and provide guidance on how to implement mentoring programs. This introductory article provides both a prelude and executive summary to the seven articles, describing the motivation, cultural context and relevant background, and presenting key findings, conclusions, and recommendations.

## The Setting: Mentoring in Low- and Middle-Income Countries (LMICs) AND EXISTING GAPS

Mentorship is the professional relationship by which the mentor, “an experienced and highly regarded, emphatic person,” guides a more junior colleague, the mentee, in developing and reassessing his/her ideas, learning and development,^1^ and substantially furthers his/her personal and professional growth. Mentorship is recognized as a deeply rooted tradition in academics^2^ and a proven path to the development of future generations of scientists. Historically, investing in the success of others has been an expected responsibility, although even accomplished scientists in high-income countries were typically not formally trained as mentors. Mentorship was performed somewhat intuitively and unofficially, with occasional reports of conflicts of interest and negative outcomes.^3^ Mentorship frameworks, tools, and programs have only emerged in the last decades.^4,5^ Regardless of potential imperfections in its implementation, mentorship can result in deep, continued friendships that evolve and mature over time. In fact, some scientists consider their relationships with mentees their most enduring contributions.

The global health revolution has accelerated the spread of mentoring cultures and practices in LMIC through the resulting exchange of academic models with high-income countries and the increasing number of LMIC students receiving advanced degrees in high-income country institutions. This has generated a growing interest from LMIC scientists to learn and introduce mentoring practices into their careers, universities, and research centers.^6^ However, formal mentoring remains an infrequent and largely unsupported practice in LMIC institutions conducting global health research^7^ and perhaps this has historically inhibited the growth of their scientists and research (Table 1). Many LMIC institutions do not yet have a strong tradition of mentoring,^8^ mentoring programs are very uncommon, very few LMIC scientists have received mentorship training, and institutions lack the resources and capacities needed to institutionalize mentoring programs and processes.^9,10^ Also, existing evidence, best practices, and norms for successful mentoring are not fit to LMICs but instead are highly biased toward the environments and resources of high-income countries,^11^ where opportunities abound and a diverse array of professionals with different backgrounds are trained, prepared, supported, and often rewarded to serve as mentors. This is a challenge for the implementation in LMIC settings: in a study in South Africa, many researchers reported mentoring increased their workload and was financially unrewarding.^12^ Opportunities to support themselves and their research are scarce and extremely competitive, and support to invest time in mentoring is often nonexistent. Having time for mentoring in LMICs is often viewed as a luxury, and therefore, the development of mentoring capacities should be coupled by institutional commitment to the implementation of mentoring programs and recognition of the critical contribution of mentoring activities. In summary, the adoption of mentoring practices in LMICs is not a straightforward process of uptake and introduction but instead a complex implementation science issue requiring adaptation of existing approaches into different academic settings^13^ (Table 1), until the needed structural change takes place.

**Table 1:** Differences between high-income and LMICs relevant to tailor mentoring efforts

Issue	High income	LMIC	Mentoring adaptation
Availability of mentors	Extensive, some trained in mentoring	Scarce, limited mentoring training	Phased implementation, train the mentor, joint-mentoring with high-income country scientists
			Group mentoring, progressive mentoring and peer mentoring*
			Mentors primarily in mid-career
Culture	Horizontal, challenging mentor’s ideas is encouraged	Tends to be hierarchical, requiring acceptance of senior’s ideas and discouraging critical thinking or challenging mentor	Establishes rules to allow respectful disagreement.
			Explicit support for diversity.
			Promotes use of appropriate and acceptable language to express differences in opinion
Relationship	Friendship, long-lasting	Paternalistic, dependence	Promotes independence and growth
Institutional resources	High	Low	Includes institutional resources in funding proposals
Institutional support for mentoring	Limited	Low or nonexistent	Phased implementation via postgraduate programs and selected, promising undergraduate scholars
			Works with institutional champions and interested research groups.
Awareness and recognition of mentoring activities	Extensive, often required for academic promotion but seldom rewarded financially or with dedicated time for mentoring activities	Limited, not usually considered for academic promotion	Institutional recognition and reward of mentoring as a key academic role, with dedicated time for mentorship activities.
			Dissemination of concept and process of mentoring among faculty and students, coupled with training
			Consideration of personal value and psychosocial support
			Advertise benefits of mentoring and success of mentors and mentees

LMIC = low- and middle-income country.

* Progressive mentors are only slightly ahead of the mentee regarding experience or experience, while peer mentors are in similar stages, although in both scenarios the mentorship relationship is mutually beneficial.

In LMICs, roles such as tutoring, “supervisorship” and “thesis advising” are often operative,^14^ and very frequently are erroneously considered and referred to as mentoring. However, these practices lack the foundations of mentoring, including maintaining effective communication, aligning mutual expectations, addressing equity and inclusion, fostering independence, and promoting professional development,^15^ in addition to the lack of providing career guidance and promoting networking. As a result, potential mentors and mentees are not necessarily aware of the implications and benefits of a mentor–mentee relationship (Table 1). A small fraction of the scientific community in LMICs, particularly younger, internationally trained doctoral-level scientists who have been exposed to a nurturing mentorship culture, tend to be more aware and are progressively introducing mentoring practices, often with little or no institutional support. Wider uptake, expansion and impact is further limited by the limited institutional awareness of and recognition for the role and value of mentorship and a reward system for mentors, as well as lack of trained mentors. Clearly, the gaps to provide adequate mentoring to the many students who would benefit are extensive, multidimensional, and go beyond currently available institutional resources.

## Cross-cultural perspectives

Low- and middle-income countries and their regions share many commonalities but also present substantial cultural differences resulting from their individual characteristics and their specific past colonial influences. The oppressive histories in many countries have contributed to research and education structures with authoritarian approaches,^13,16^ which in turn are additional obstacles for effective mentoring. Culture distills a deeply rooted respect for hierarchy and seniority (Table 1), which is echoed in medicine, academics, and research,^17^ as well as strict formality in communication and dialog to the point that challenging the opinions of a senior scientist or faculty can often be considered offensive or inappropriate.^16^ The formal addressing of peers and superiors by their titles and ranks instead of first names continues to be considered a sign of respect in LMICs, even in regions such as South America where warm, close interpersonal relationships and informal interactions are the social norm. This can represent a barrier to the development of a strong mentor–mentee relationship, preventing the trust that can enable questioning or disputing of the mentor’s position or views.^18^ In addition, the absence of such a close link may prevent the creation of a personal bond that frequently can make mentorship relationships last beyond specific training periods. Verticality and formality is present in varying degrees across continents and countries, which promotes paternalism and limits the ability of “mentoring up,” a process through which mentees are empowered to direct the mentoring relationship, and thus, places equal or greater emphasis on the mentee’s contribution to the mentoring relationship.^15^ The scarcity of resources and opportunities adds to this, as the success of the mentee can be erroneously perceived by mentors as increased competition and failure. Furthermore, remnants of colonial master–servant beliefs may prevent mentors from understanding that mentorship implies a greater shared power between the mentor and mentee instead of the mentor alone having all the power.

Approaches to punctuality, the importance of rules and regulations, and the meaning of deadlines and commitments often differ between LMICs and high-income countries and could limit mentoring success. In addition, evaluations in LMIC settings are often very strict and focus primarily on “academic” performance without consideration of professionalism, ethics/integrity, and work-life balance; topics that are often addressed in formal mentoring programs. Another practice that varies across cultures is grade inflation. Low- and middle-income country mentees often do not benefit from the widespread practice of grade inflation that takes place in some high-income countries,^19^ which can result in less enthusiastic letters of support and recommendation from both high-income and LMIC mentors, leading to lower chances of success and discouragement to advance in academics. All these factors can also influence how collaboration shapes between scientists, reduce the depth and diversity of mentoring, and in some cases exacerbate territoriality, reducing the chances of scientific collaboration.

Several of the issues described in these mentoring articles are rapidly evolving and should be assessed in the specific LMIC contexts, where mentoring will be implemented. The age, gender, culture, beliefs, place of training, and diversity of LMIC students, research leaders, principal investigators, and senior scientists should also be considered. For instance, the mean age of mentors tends to be higher and female participation more limited than in high-income countries,^20^ particularly at the highest levels of decision making. Some countries are making important headway in this area, and research and training support tends to prioritize younger candidates.^21^ However, reentry opportunities for women or older scientists with family responsibilities tend to be scarce or nonexistent, limiting their advancement while also amplifying disparities, reducing the mentor pool, and increasing the burden of existing mentors.

## Institutionalization of Mentorship in LMICs

We propose the long-term goal of creating a local “identity” of mentorship within the cohesive social fabric of LMICs and developing models that build on local strengths, while dealing with factors such as hierarchy and social structure. The introduction and strengthening of mentorship in the specific cultural, economic, and structural settings of LMICs should have aims and approaches pragmatically compatible with available resources and institutional support (Table 1). A progressive and phased implementation science approach will require customization to the local institutional setting, pilot programs, and eventually scale-up efforts once LMIC-specific best practices and lessons learned are clear, and impact indicators are well defined. Just as in high-income settings, institutional mentoring efforts at LMICs will limited by available resources and will take time to initiate, scale-up and become self-sustaining. In the meantime, closely monitored and evaluated pilot introductions in individual research groups, departments, or academic programs at a smaller scale can serve as proof of concept. These initial efforts, some already underway, should produce diffusion of innovations and experiences on how to best advocate for the implementation of institutional-level mentoring programs in the future. Another key outcome of LMIC-mentoring programs should be principles, best practices, and evidence-based guidelines for others to replicate and expand successful efforts and models. Mid- or senior-career scientists with extensive exposure to international practices of mentoring should play key roles in leading initial mentoring programs (Table 2). Efforts to train and “mentor” the mentors will be needed and represent a key task of the global health academic community. Table 2 presents a few key recommendations for specific stakeholders in this endeavor. Together, these elements will eventually help to shape the identities of mentorship in LMICs.

**Table 2:** Recommendations for the strengthening of mentoring in low- and middle-income countries

Institutions	Mentors (faculty/scientists)	Mentees (students)
Provide a policy framework	Complete formal and informal training and commit to improving mentoring skills	Complete research integrity training and learn how to work effectively with mentors
Formally recognize the value/role of mentoring in academic and research activities	Introduce mentoring within research groups and graduate/professional degree programs	Learn about mentee roles, mentee–mentorship experiences and choose committed mentors
Acknowledge importance of investing in future scientists and in research-enabling environments	Promote values and independence among mentees and stimulate scientific debate and disagreement	Be willing to listen to and engage with mentors
Promote mentorship culture and altruism, and research integrity	Support diversity of ideas and inclusivity, and be willing to listen and engage with trainees without judgment	Proactively contribute to define expectations and goals
Acknowledge and reward the best mentors	Promote mentees’ growth into independence, encourage work-life balance	Assess progress and help to improve the experience when expectations are not met
Invest resources in training mentors and compensate them for time spent mentoring	Evaluate the outcomes and impact of mentoring efforts.	

Low- and middle-income countries can take advantage of unique opportunities to implement mentoring programs, such as the earlier engagement of undergraduate students in research compared with high-income countries and the large cohorts of LMIC researchers returning home as potential mentors after degree training in international programs. An important foundation of ongoing international research training opportunities is described in the next section. Developments in Peru, Uganda, and Kenya are only a few. In addition, new treatment and prevention opportunities can support mentoring of young researchers, such as the roll out of community-based HIV pre-exposure prophylaxis and the emerging focus on the non-communicable diseases epidemic, among others. These can be excellent vehicles for building mentorship systems.

## Fogarty-sponsored mentoring workshops

Historically, LMICs have been the focus of substantial research capacity-building investments, ranging from the Rockefeller Foundation's International Clinical Epidemiology Network^22^ to the U.S. National Institutes of Health Fogarty International Center’s (FIC) Global Infectious Diseases programs, Global Health Program for Fellows and Scholars,^23^ and Medical Education Partnership Initiative.^24^ Other recent efforts include the Wellcome Trust’s African Institutions Initiative^25^ and Brazil’s Science without Borders,^26^ in addition to activities supported by the U.S. Centers for Disease Control and Prevention, and the U.S. Agency for International Development. None of these investments, however, has specifically addressed the need for local mentoring models or approaches in LMICs. Cole describes a few examples of mentoring experiences and programs in high-income and LMICs, highlighting multiple research gaps and the need for greater evaluation and systematic assessment.^27^

To help address these needs, the FIC Global Health Program for Fellows and Scholar consortia members partnered to offer five regional “Mentoring the Mentors in Global Health Research” workshops at LMIC institutions. Each 2-day workshop was led by experienced faculty from each region in collaboration with senior U.S. faculty affiliated with the FIC-supported consortia and provided participants with the definitions, methods, and tools to become effective mentors in LMIC settings. Workshops included between 19 and 37 mid- and senior-level scientists primarily from universities and other global health research institutions. The first two workshops were conducted back-to-back in May 2013 in Lima, Peru, for South American training sites and in June 2013 in Mombasa, Kenya, for East African sites. The third workshop was held in November 2014 in Bangalore, India, for the South Asian sites and the fourth workshop took place in Johannesburg, South Africa, in March 2016 for southern African sites. A fifth workshop was conducted for West and Central Africa in Accra, Ghana, in May 2018. All five workshops received positive reviews addressing a much-needed training gap.

Given the success of the first four workshops, in 2017 we held a senior-level technical advisory meeting on global health mentoring at LMIC institutions. Leaders from the first four workshops, along with the principal investigators of the six FIC Global Health Program for Fellows and Scholars consortia and staff from FIC gathered to discuss and plan how to best support the development, sustainability, and productivity of strong mentorship programs in global health in LMIC institutions. The goal for this *American Journal of Tropical Medicine and Hygiene* (*AJTMH*) series of articles is to help herald in a new era of increased mentoring in LMICs that leads to advancement of global health research and practice around the world.

## The *AJTMH* LMIC mentoring special issue

This special issue addresses the challenge of implementing mentoring programs in LMICs and provides guidance on how to adapt mentoring practices used in high-income countries to the settings and cultural practices of research and academic institutions in resource-limited settings. These publications are directed at scientists, institutional leaders, administrators, and trainees in LMIC institutions in collaboration with partners in high-income countries interested in expanding mentorship at LMIC institutions to advance global health research. The articles can also serve as a reference guide for LMIC institutions to develop strategies, approaches, and programs to support mentorship across institutional units, including departments, schools, and colleges, and to select priority mentoring practices for implementation supported by a strong, LMIC-specific evidence base.

The recommendations presented in the articles follow four principles highlighting the philosophy of this special issue. First, LMIC scientists led content development as first or senior authors, for an intended audience of LMIC scientists and their high-income country partners in mentoring efforts, as well as LMIC institutions in resource-limited settings. Second, all content was developed exclusively to guide the implementation of mentoring programs and activities in LMICs and to address LMIC-specific gaps. Third, embracing diversity is paramount, as culture, settings, resources, and opportunities for mentoring differ substantially across LMICs. A one-size-fits-all approach is neither viable nor desirable, and implementation will demand customization to each institution, program, or group interested in advancing mentoring. Fourth, a rigorous evidence- and best practices–based approach to mentoring in LMICs is critically needed, and these articles are only the first step in the process. Further refinement of the proposed recommendations is warranted.

This overview piece serves both as a prelude to the seven detailed articles, describing the overall motivation and questions and issues each paper addresses, as well as an executive summary, presenting the key findings, conclusions, and recommendations for next steps. The seven other articles in this special issue address specific aspects of the introduction and strengthening of mentorship practices in LMICs. Shailendra Prasad and others^28^ propose a conceptual framework of mentoring tailored to LMIC settings and David Hamer and Laetitia Rispel et al.^29^ identify critical, technical, and cultural competencies required for mentoring of LMIC scientists. Monica Gandhi, Craig Cohen, and others compare the barriers and facilitators of mentoring identified during the LMIC mentorship workshops sponsored by the FIC Global Health training programs that started this initiative. The evolution of mentorship efforts after the workshops is presented via case studies by Emilia Noormahomed, Craig Cohen et al.,^30^ describing existing gap and mentoring needs in various institutions in Africa, Asia, and South America. A framework for the evaluation of mentoring programs, including domains and metrics, is proposed by Benjamin Chi, Tony Raj, and others,^31^ whereas common issues related to ethics and research integrity in LMICs are examined by Elizabeth Bukusi, Joseph Zunt, and others^32^ via four selected case study scenarios. The collective knowledge of available mentoring toolkits relevant for LMICs and related gaps is discussed in a scoping review conducted by Bhakti Hansoti and others.^33^ All authors have attempted to link the contents of each of these articles, recognizing that over time, mentoring practice and implementation in LMICs will lead to more cohesive and updated versions of these tools.

## Conclusion

The advancement of global health research demands sustained career development opportunities for LMIC scientists that can only be attained via the implementation and dissemination of culturally compatible mentoring practices. Institutional resources and local academic and cultural factors should guide the phased implementation of tailored mentoring activities and programs for each setting, with planned, periodic evaluation of progress. Low- and middle-income country institutions also need to support existing mentors and train additional ones, while mentees can contribute to prevent overburdening the few trained mentors available, by playing an active role in the operational efforts of mentoring programs via progressing and peer mentoring. We hope this special issue will become part of the foundation of LMIC-specific mentoring approaches around the globe.
